# Supplementary material for: MetaRibo-Seq measures translation in microbiomes
Source: Nat Commun. 2020 Jun 29;11:3268. doi: 10.1038/s41467-020-17081-z (PMC7324362; doi:10.1038/s41467-020-17081-z)
Supplement: Supplementary file 10 — Supplementary Data 7 [file 41467_2020_17081_MOESM10_ESM.zip › File2/Confidence_VeryHigh_Taxonomy/337749_out.krona.html]

Javascript must be enabled to view this page.

members
magnitude
magnitudeUnassigned
count
unassigned
taxon
rank

337749\_out

12

12
superkingdom
2

12
phylum
976

12
class
200643

order
171549
12

11
171552
family

11
genus
838

1

SRS019808\_contig\_number\_20283
species
2079531

1

SRS049959\_contig\_number\_15964
species
1262932


SRS044535\_contig\_number\_17237SRS146764\_contig\_number\_33049
2
species
1262937

1263102
species

SRS023914\_contig\_number\_4704
1

6

SRS022609\_contig\_number\_41693SRS043667\_contig\_number\_424SRS049995\_contig\_number\_29631SRS053356\_contig\_number\_47058SRS1041031\_contig\_number\_21138SRS1041140\_contig\_number\_21603
species
165179

family
815
1

genus
816
1

species
371601

SRS022609\_contig\_number\_15056
1
